# AI Integration in Spanish Undergraduate Medical Education: National Cross-Sectional Study

**DOI:** 10.2196/88511

**Published:** 2026-06-08

**Authors:** Ana Enériz Janeiro, Karina Pitombeira Pereira, Julio Mayol, Javier Crespo, Fernando Carballo, Juan B Cabello, Manuel Ramos-Casals, Bibiana Pérez Corbacho, Juan Turnes

**Affiliations:** 1 IDARA Research Group, Galicia Sur Research Institute (IIS Galicia Sur), Vigo, Spain; 2 MedicineAI, Madrid, Spain; 3 Hospital Clínico San Carlos, Madrid, Spain; 4 Universidad Complutense de Madrid, Madrid, Spain; 5 Instituto de Investigación Sanitaria del Hospital Clínico San Carlos, Madrid, Spain; 6 Instituto de Investigación Marqués de Valdecilla, Santander, Cantabria, Spain; 7 Universidad de Murcia, Murcia, Spain; 8 Critical Appraisal Skills Programme: CASP Spain, Alicante, Spain; 9 Postgraduate Degree Program in Artificial Intelligence in Medicine, Universidad de Cádiz, Cádiz, Andalusia, Spain; 10 Department of Gastroenterology and Hepatology, Complejo Hospitalario de Pontevedra, Mourente, s/n, Pontevedra, Galicia, 36071, Spain, +34 986 80 09 07

**Keywords:** artificial intelligence, AI, generative artificial intelligence, medical education, curriculum, Spain

## Abstract

**Background:**

Artificial intelligence (AI) is reshaping clinical practice and redefining the competencies future physicians will need. International bodies, such as the Association of American Medical Colleges, have called for structured AI training in medical curricula. Despite growing international consensus, no systematic nationwide evaluation had been conducted in Spain prior to this study.

**Objective:**

This study aimed to characterize the presence, type, and curricular features of AI-related training across all Spanish universities offering an official medical degree and to assess differences by institutional ownership and geographic region.

**Methods:**

This cross-sectional study was conducted from July to September 2025. Universities were the unit of analysis. A census of all institutions offering an officially recognized medical degree was obtained from the Register of Universities, Centers and Degrees; all 52 eligible institutions were included. Publicly available curricula and course guides for the 2025‐2026 academic year were reviewed by 2 independent researchers and validated by an external evaluator. Courses were classified as (1) a specific AI course (AI as primary topic, accounting for >50% of syllabus), (2) an AI-similar course (a digital health or biomedical informatics course referencing AI as secondary content), or (3) not AI-related training. Course-level variables included ownership (public or private), region, status (compulsory or elective), European Credit Transfer and Accumulation System (ECTS) credits, academic year, and department. All analyses were descriptive. Potential sources of bias were addressed through predefined classification criteria, duplicate independent extraction, and external dataset verification.

**Results:**

Of 52 universities, 36 (69.2%) were public and 16 (30.8%) were private. A total of 10 (19.2%) institutions offered at least one specific AI course; 6 (11.5%) included an AI-similar course. Overall, 16 (30.8%) universities had incorporated AI in some form; 36 (69.2%) institutions had not incorporated AI. Rates were similar for public (7/36, 19.4%) and private institutions (3/16, 18.8%). Identified courses ranged from 3 to 6 ECTS credits, representing an average of 1.17% of the 360-credit degree; most were elective. Only the University of Jaén offered a compulsory course with AI content. Marked regional disparities were observed: Andalusia led with 5 of 9 (55.6%) universities offering a specific AI course, while 10 autonomous communities had no universities with any AI-related training.

**Conclusions:**

This study delivers the first census-based, reproducible, national assessment of AI integration in Spanish undergraduate medical education. Unlike prior work focused on individual programs or nonstandardized definitions, we applied a consistent taxonomic framework reusable for longitudinal monitoring and international benchmarking. Findings reveal a heterogeneous, predominantly elective, and low-weight curricular landscape with striking interregional inequities. These results inform curriculum reform, accreditation standards, and faculty development priorities and support the establishment of minimum national competency standards and systematic monitoring to ensure equitable AI literacy among future physicians in Spain.

## Introduction

Artificial intelligence (AI) has emerged as a transformative technology in the field of health care, revolutionizing everything from medical diagnosis to hospital management and biomedical research [1]. This technological revolution is not only transforming current clinical practice but also redefining the competencies that future health care professionals will need to practice medicine effectively in the coming years [2].

The integration of AI into medical curricula is globally recognized as a priority, underscoring the need to prepare future physicians with the technical and ethical competencies required to ensure the responsible use of these technologies. Moreover, there is an urgent need to develop structured curricular frameworks that systematically incorporate AI education into medical schools [3].

However, the translation of these recommendations into concrete curricula has been uneven and fragmented. The advent of generative AI adds urgency, as it amplifies both the educational potential (eg, support for writing and supervised reasoning) and the risks (hallucinations, data leakage, and authorship attribution), making it essential to teach strategies for verification, documentation, and boundaries of use. The lack of AI training is also acknowledged by the students themselves. Global and regional surveys show broad support for its teaching, calling for its inclusion in curricula while also expressing concern about ethical issues, the physician-patient relationship, and its impact on specialty choice [4,5]. In a study conducted with medical students in Spain, 83% considered it essential to acquire knowledge of AI for their future professional practice [6].

To address this need, several countries and organizations have begun implementing systematic initiatives to incorporate AI into medical education. In Canada, curricular proposals and needs assessments have been developed to identify key competencies in areas such as ethics, legal aspects, clinical application, and collaborative work [7,8]. In the United States, several medical schools have advanced the formal integration of AI into their curricula, with prominent examples including the Icahn School of Medicine at Mount Sinai, the latter being a pioneer in its cross-curricular incorporation [9]. In addition, international studies involving medical students and faculty consistently indicate that AI training remains limited and urgently call for curricular updates to address the digital transformation in health care [4,10,11].

The Spanish context presents relevant particularities: 52 medical schools, a 360-ECTS (European Credit Transfer and Accumulation System) degree program, and quality assurance frameworks that both enable and require transparent curricular structures. Despite the proliferation of isolated initiatives, to the best of our knowledge, there is no national, systematic, and reproducible characterization of how AI is being incorporated into curricula, what educational weight it receives (in ECTS), whether it is compulsory or elective, and to what extent it includes specific content on generative AI.

Recent syntheses highlight that, despite AI’s growing relevance to clinical decision-making and health system workflows, undergraduate medical education remains uneven and fragmented globally. A scoping review mapping the use of AI across medical education settings found that, while AI applications are proliferating in areas such as clinical reasoning support and automated assessment, their integration into formal curricula remains largely ad hoc and driven by individual institutional initiatives rather than coordinated policy. Persistent implementation barriers (including limited faculty capacity, unclear competency targets, insufficient institutional support, and the absence of consensus on what should be taught and how learning should be assessed) continue to hinder systematic adoption. These findings underscore the need for national-level mapping studies, such as this one, to identify gaps and inform evidence-based curricular reform [12].

Although several curriculum frameworks and educational initiatives have been proposed to define core AI competencies for medical learners, approaches still vary widely in terminology, depth, and evaluation. Some frameworks emphasize foundational data science concepts, others prioritize clinical decision support skills, and still others focus on ethical and regulatory dimensions, with little overlap in learning objectives or assessment strategies. This heterogeneity limits comparability across institutions and hinders the establishment of benchmarks and minimum standards that could ensure baseline AI literacy for all medical graduates. The lack of a shared taxonomic language for classifying AI content in medical curricula makes it particularly difficult to compare findings across countries or track progress over time, a gap that this study aims to address by proposing a reproducible classification framework [13].

The emergence of generative AI has heightened the urgency for structured training in medical schools. Tools such as ChatGPT (OpenAI), Gemini (Google Inc), and other large language models are already being used by medical students for study support, clinical reasoning, and academic writing, often without formal institutional guidance. This raises concerns about hallucinated content, data privacy, academic integrity, and uncritical reliance on AI-generated outputs. Therefore, medical schools need not only to teach the fundamentals of AI but also to develop governance frameworks, ethical guidance, and practical instruction on the safe and responsible use of generative AI alongside monitoring mechanisms to track curricular adoption and inform institutional policy. Against this background, understanding the current state of AI education in a national context becomes essential for identifying priority areas for intervention [14].

The primary objective of this study was to provide the first analysis of the current state of AI integration in undergraduate medical education programs in Spain, based on reproducible criteria and supported by an open dataset for verification and reuse. Specifically, we aimed to characterize the presence, type, and curricular features of AI-related teaching across Spanish universities offering a medical degree and to assess differences by university ownership (public vs private) and geographic region. We hypothesized that AI-related training would be limited in scope, predominantly elective, and unevenly distributed across institutions.

## Methods

This study was prepared in accordance with the STROBE (Strengthening the Reporting of Observational Studies in Epidemiology) statement for cross-sectional studies, and the corresponding checklist was used to ensure complete reporting of study design, setting, variables, data sources, bias, and statistical methods (Checklist 1).

### Study Design

This was a cross-sectional study conducted between July and September 2025 to analyze the integration of AI education in undergraduate medical programs in Spain.

### Setting

A census of all Spanish universities offering an official degree in medicine was conducted using the “Register of Universities, Centers and Degrees (Registro de Universidades, Centros y Títulos)” (RUCT) [15], following the search parameters specified in Table 1.

**Table 1.** Consultation on medical degrees offered in Spain: Register of Universities, Centers and Degrees (Registro de Universidades, Centros y Títulos; RUCT)^a^.

Search field	Search selection
University	All
Degree name	Medicine
Academic level	Bachelor’s degree (*grado*)
Field	Health sciences
Area	Medicine and dentistry
Status	All
Historical search	No

a Data collected from July to September 2025 (academic year: 2025‐2026). The table lists the query fields and selections applied in the RUCT to retrieve eligible institutions.

### Participants and Unit of Analysis

The unit of analysis was the university. This registry currently lists 52 universities offering a degree in medicine in Spain; the RUCT database reflects a 2021 update, and all institutions were independently confirmed as actively offering the program for the 2025‐2026 academic year through direct portal access (last verified on September 4, 2025). A complete list of the 52 included universities, with RUCT identifiers and source URLs for verification, is provided in Multimedia Appendix 1. Universities were eligible if they (1) offered an officially recognized medical degree program for 2025‐2026 and (2) had publicly accessible curricular information sufficient to assess whether AI-related training was present in the curriculum (degree plan or course guides). No restrictions were applied based on ownership or region.

### Variables

The search strategy was based on the selection of institutions currently offering an active degree in medicine. The institutional websites of each university were reviewed to identify information on curricula, courses, and AI-related competencies within the medical degree for the 2025‐2026 academic year. This study relied exclusively on publicly available information from official websites and public curricular documents, including study plans, course programs, and teaching guides.

A university was considered to offer a specific AI course if the Spanish term for “artificial intelligence” (“*inteligencia artificial*”) or “AI” (“*IA”*) appears in the course title and/or if more than 50% of the content is dedicated to AI.

The existence of courses that might include content on AI, even if not explicitly stated in the title, was also examined. The Spanish keywords used for this selection were “new technologies” (“*nuevas tecnologías”*), “informatics” (“*informática”*), “communication” (“*comunicación”*), “digital media (“*medios digitales”*),” or “digital health” (“*salud digital”*). Thus, a university is considered to offer a course “similar to AI or appearing in competencies” when AI accounts for less than 50% of the course syllabus and/or when reference is made to the keywords. Universities are considered to have no AI courses when they do not include any course on AI or similar to AI.

The main outcome was the presence of AI-related training in the medicine curriculum, operationalized into mutually exclusive categories based on course content, as follows: specific AI courses (the course explicitly addresses AI in medicine or health care as a primary topic), AI-similar courses (courses focusing on computational, statistical, or digital health competencies closely related to AI, eg, data science and bioinformatics, but without explicitly positioning AI as the main subject), and non-AI-related training (there was no evidence of AI-related content in the degree plan or available course guides).

For each course, the following information was collected: university, region, type (public or private), title, status (compulsory or elective), training credits, academic year in which it is offered, department (or alternatively, the specialty of the faculty teaching it), and main course content.

### Data Sources and Measurement

This study relied exclusively on publicly available information from official university websites and public curricular documents, including study plans, course programs, and teaching guides. To ensure the quality and accuracy of the collected data, each data entry was independently verified by 2 researchers and validated by an external evaluator not involved in the data collection process.

### Bias

We anticipated potential sources of bias typical of web-based curriculum assessments: (1) information bias due to incomplete or outdated public documentation, potentially underestimating AI-related training; (2) misclassification bias due to heterogeneous terminology across universities (AI content embedded under non-AI labels); and (3) selection bias at the variable level when specific course attributes (eg, department or academic year) were not publicly reported. To mitigate these risks, we applied predefined classification criteria (including the >50% rule), used duplicate independent extraction, and conducted external verification of the compiled dataset.

### Study Size

Study size was determined a priori by a census of all eligible universities in Spain offering an official medical degree in 2025‐2026. The final analytic sample comprised 52 universities.

### Statistical Methods

All analyses were descriptive. We summarized the number and proportion of universities that offered a specific AI course, an AI-similar course, or no AI-related training, and we described the characteristics of identified courses—such as whether they were compulsory or elective, their ECTS credits, the academic year or level, and the responsible department—whenever this information was available. Given the census-based and primarily descriptive aim of the study, we did not fit adjusted models to control for confounding. To explore heterogeneity, we presented results stratified by university ownership (public or private) and by autonomous community or region. When specific variables were not publicly reported (eg, department, academic year or level, or ECTS credits), they were coded as missing and analyses were conducted using the available information, with denominators varying accordingly. Because we assessed the full population of eligible programs rather than a sample, methods accounting for a sampling strategy were not applicable, and no formal sensitivity analyses were performed.

To examine geographic variation, we conducted a stratified descriptive analysis by autonomous community. For each community, the denominator was the number of universities offering an official medical degree, and we calculated the proportion of universities that offered at least one specific AI course and an AI-similar course, as defined in the study taxonomy. Communities with no medicine programs (denominator=0) were not assigned estimates.

### Ethical Considerations

This study analyzed exclusively publicly available curricular information from official university websites and institutional documents. No individual-level human data were collected, and no surveys, interviews, or interactions with human participants were conducted. In accordance with Spanish regulations governing research that relies solely on publicly accessible, nonpersonal data, formal ethics committee review was not required, and the study was exempt from institutional review board approval. This exemption is consistent with the provisions of Spanish Law 14/2007 on Biomedical Research [16], which applies to studies involving human subjects or their biological samples. As this study did not involve human participants or personal data, informed consent was not applicable; no primary data collection from individuals took place, and therefore no consent process was required. All data used in this study were obtained from publicly accessible institutional sources. No personally identifiable information was collected at any stage; the dataset contains only institution-level variables (university name, region, and course characteristics), and no individual-level data were gathered, stored, or processed. Therefore, the study poses no risk to the privacy or confidentiality of any individual. No human participants were involved in this study; therefore, no compensation was provided or applicable. No images containing identifiable individuals are included in this manuscript or its supplementary materials.

## Results

A total of 52 universities offering an official medical degree were identified through the RUCT. We then carried out systematic verification using publicly available institutional sources, university websites, and course guides to confirm eligibility and extract curriculum-level and course-level variables. All 52 eligible universities were included in the analysis, and no institutions were excluded.

### Scope and Distribution

The analysis included a total of 52 Spanish universities offering a degree in medicine, representing all institutions with officially recognized programs in Spain for the 2025‐2026 academic year. Of these universities, 36 (69.2%) were public institutions and 16 (30.8%) were private institutions.

The territorial distribution showed a concentration of medical schools in certain regions. Madrid led with 10 (19.2%) universities, followed by Andalusia with 9 (17.3%) and Catalonia with 8 (15.4%). The Valencian Community had 6 (11.5%) universities, while the Canary Islands had 3 (5.8%) institutions. The remaining autonomous communities each had between 1 (1.9%) and 2 (3.8%) universities, revealing a heterogeneous distribution that reflects both population density and the historical development of medical education in each territory.

### Distribution of AI Education

Of the 52 universities analyzed, 10 (19.2%) offer courses specifically dedicated to AI in medicine (Table 2). Another 6 (11.5%) universities include an AI-similar course within broader courses or mention AI-related competencies in their learning objectives (Table 3). Therefore, considering both universities with specific AI courses and those with AI-similar courses, a total of 16 (30.8%) institutions have incorporated AI into medical education in some form, whereas 36 (69.2%) institutions have not yet included explicit AI content in their medicine curricula.

**Table 2.** Universities with specific artificial intelligence (AI) courses in the medical degree program in Spain^a^.

University	Type	Region	Course	Status	ECTS^b^ credit	Year/level of medical degree program	Department	Generative AI inclusion
Autonomous University of Barcelona	Public	Catalonia	Artificial intelligence and health	Elective	3	Third	Surgery	No
Complutense University of Madrid	Public	Madrid	Practical application of generative artificial intelligence	Elective	3	First and second	Radiology, Rehabilitation, and Physiotherapy	Yes
Complutense University of Madrid	Public	Madrid	Big data and artificial intelligence in medicine	Elective	3	Third and sixth	Medicine	No
Fernando Pessoa University, Canary Islands	Private	Canary Islands	Big data and artificial intelligence in medicine	Elective	3	—^c^	—	—
University of Huelva	Public	Andalusia	Application of big data and artificial intelligence in medicine	Elective	3	Fourth	—	—
Loyola Andalusia University	Private	Andalusia	Artificial intelligence and healthcare	Elective	3	Fourth	—	—
University of Lleida	Public	Catalonia	Artificial intelligence in medicine	Elective	3	First	Basic Medical Sciences	No
Camilo José Cela University	Private	Madrid	Fundamentals of artificial intelligence in healthcare	Elective	3	Third	—	Yes
University of Córdoba	Public	Andalusia	Documentation and artificial intelligence in medicine. history of medicine	Basic	6	First	Medical and Surgical Sciences	No
University of Almería	Public	Andalusia	Medical informatics	Elective	3	Fourth	Informatics	No
University of Jaén	Public	Andalusia	New clinical and biomedical information technologies	Compulsory	3	Second	Microbiology	No

a Cross-sectional census of 52 Spanish universities offering an official medical degree (academic year: 2025‐2026); data collected from July to September 2025.

b ECTS: European Credit Transfer and Accumulation System.

c Information not publicly available or not verifiable from publicly accessible institutional sources.

**Table 3.** Universities with courses categorized as artificial intelligence (AI)–similar course in medical degree programs in Spain^a^.

University	Type	Region	Courses	Status	ECTS^b^ credit	Year/level of medical degree program	Department	AI content detail
University of Vic - Central University of Catalonia	Private	Catalonia	Information and communication technologies and health	Elective	5	First and second	—^c^	It mentions AI in the objectives of existing courses, and the university has a specific AI professorship
Alfonso X El Sabio University	Private	Madrid	Digital health	Elective	6	Sixth	—	Among the contents detailed are AI in value-based health care; AI-based decision support tools; relevant data in the health care setting and methods for acquiring, preparing, and storing them; data mining; natural language processing; machine learning; and success stories of intelligent systems, such as IBM Watson, including associated challenges, barriers, and facilitators
University of Extremadura	Public	Extremadura	Applied medical informatics	Elective	6	Fifth	Department of Computer Systems and Telematics Engineering, Languages and Computer Systems	Contents include fundamentals of informatics, introduction to medical informatics, search and management of health information, communication and dissemination of health information, and health information systems
Rovira i Virgili University	Public	Catalonia	New Technologies and data management	Compulsory	3	Third	—	The course includes management and analysis of health data with advanced technological components
University of Oviedo	Public	Asturias	Pathological anatomy	—	—	—	—	—
University of Oviedo	Public	Asturias	Fundamentals of surgery	—	—	—	—	AI is mentioned in Topic 8 (“New challenges and future of surgery”), specifically in Session 31 (“Telesurgery: robotics, virtual reality, and artificial intelligence”)
King Juan Carlos University	Public	Madrid	Introduction to medicine: medical information and documentation	Compulsory	6	First	Medical Specialties and Public Health	Module I includes information management and clinical research questions (highlighting Unit 7 on “Fundamentals of evidence-based medicine”); formulating problems and questions from the patient to the clinician-researcher; the DEPTh model; questions and levels of training from the patient to the researcher; and AI-based tools for evidence-based medicine, specifically generative AI tools (GPTs, ChatGPT, Ellicit, and others), for reading, analysis, and writing

a Cross-sectional census of 52 Spanish universities offering an official medical degree (academic year: 2025‐2026); data collected from July to September 2025.

b ECTS: European Credit Transfer and Accumulation System.

c Information not publicly available or not verifiable from publicly accessible institutional sources.

Information on some course-level characteristics (eg, department and academic year or level) was not publicly available for all courses and was therefore reported as not available.

By type of institution, among the 36 public universities, 7 (19.4%) offered specific AI courses, whereas among the 16 private universities, 3 (18.8%) included AI courses in their curricula (Table 4).

**Table 4.** Distribution of artificial intelligence (AI) courses by type: specific AI, AI-similar course, or no AI training^a^.

University type	Specific AI course, n (%)	AI-similar course, n (%)	AI training, n (%)
Public (n=36)	7 (19.4)	4 (11.1)	25 (69.4)
Private (n=16)	3 (18.8)	2 (12.5)	11 (68.8)
Total (n=52)	10 (19.2)	6 (11.5)	36 (69.2)

a Census of 52 universities in Spain (academic year: 2025‐2026); data collected from July to September 2025. Values are counts and percentages of universities offering at least one specific AI course, AI-similar content, or no AI-related training in publicly available curricular documentation.

**Figure 1.**
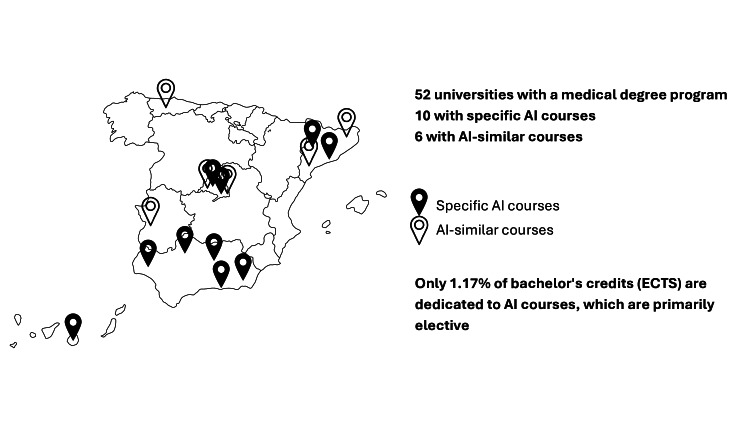
Territorial distribution of universities with artificial intelligence (AI) courses in the medical degree program in Spain. ECTS: European Credit Transfer and Accumulation System.

The territorial analysis revealed a marked disparity among autonomous communities. Andalusia led, with 5 (55.6%) of its 9 universities offering specific AI courses. It was followed by Catalonia, with 2 (25%) of 8 universities, and Madrid, with 2 (20%) of 10 universities. In the Canary Islands, 1 (33.3%) of the 3 universities had incorporated a specific AI course. An AI-similar course was additionally identified in Catalonia, Madrid, Extremadura, and Asturias. By contrast, 10 regions—Aragon, Balearic Islands, Cantabria, Castile-La Mancha, Castile and León, Valencian Community, Galicia, Murcia, Navarra, and the Basque Country—did not have universities that had integrated any form of AI-related training into their medicine curricula (Figure 1).

### Characteristics of AI Education

Among the universities that included specific courses on AI in their curricula, most of them were offered as electives. The only exceptions were the University of Jaén, which offered a compulsory course, and University of Córdoba, which included a core course (Table 2). The course at the University of Jaén represented a unique case, as it was the only institution that offered a compulsory course with AI content within the medical degree. Although the course did not explicitly mention AI in its title, “New Clinical and Biomedical Information Technologies (“*Nuevas Tecnologías de la Información Clínica y Biomédica*),” its syllabus explicitly incorporated competencies related to this field, making it the only identified case of mandatory curricular integration.

### ECTS Credits

The analysis of the credit load dedicated to AI in the medical degree revealed that the specific AI courses identified ranged between 3 and 6 ECTS credits (Table 2). In relation to the total of 360 credits required for the degree, AI training constituted, on average, 1.17% of the medical curriculum. Nevertheless, since most of these courses were offered as electives, they were not included in the compulsory credit load, and not all students enrolled in them. Consequently, the actual weight of AI training within the degree program may be regarded as even smaller. Complutense University of Madrid showed the highest dedication with 6 credits, while other institutions generally offer 3-credit courses. In some cases, AI content was included within broader courses on medical informatics or health technologies, which made it difficult to quantify its exact weight.

## Discussion

### Principal Findings

Despite progress in incorporating AI into clinical medicine, its presence in medical curricula in Spain remains very limited and heterogeneous. Most institutions that have included it do so through elective courses of low academic weight (1.17% of the 360 ECTS credits), with approaches ranging from specific courses to the inclusion of AI within transversal competencies but with no common framework or clearly defined learning objectives—a pattern consistent with the global gap documented in recent international research [13,14]. Available data from Spanish medical students corroborate this assessment: in a cross-sectional survey across 192 faculties, the Spanish subset (74 students from 5 universities) reported a lack of AI courses, with 74% describing limited knowledge and 80% expressing unpreparedness to apply AI in professional practice [17]. Bridging this gap calls for coordinated policy action, including the transition of elective AI courses into core or compulsory content, dedicated faculty training programs, and a national framework of competencies analogous to those promoted by the Association of American Medical Colleges and the General Medical Council [18,19].

The territorial analysis revealed a marked disparity: while Andalusia led with 5 (55.6%) of 9 universities offering a specific AI course, 10 autonomous communities had no universities offering any AI-related training. This pattern, consistent with reports from other countries [4], reinforces the need for national strategies to reduce geographic inequity. Structural factors specific to the Spanish context help explain the pace of adoption: decentralized governance across 17 autonomous communities, the absence of explicit AI requirements in national accreditation standards, and limited faculty capacity to design and deliver AI curricula together slow progress [12,13].

Internationally, institutions in the United States such as the Icahn School of Medicine at Mount Sinai and the Stanford Center for Artificial Intelligence in Medicine and Imaging have pioneered the formal integration of AI into medical education [20,21]. In Europe, countries including Germany, Belgium, and the United Kingdom have launched pilot programs to assess essential AI competencies and develop adapted curricular frameworks [22-24]. Spain’s position, characterized by the limited and predominantly elective nature of its current provision, underscores how much ground remains to be covered.

The case for reform is amplified by converging pressures. Spain’s aging population and growing burden of chronic disease demand optimization of health care resources [25], and while AI offers tools to improve diagnostic accuracy, personalize treatments, and reduce clinical errors [26], realizing these benefits requires professionals capable of navigating the ethical and interpretative challenges involved [27]. The rapid evolution of generative AI compounds this urgency: models such as Google’s MedLM are already deployed in clinical settings [28], and multimodal systems are achieving high accuracy in combined medical tasks [29], making curricular adaptation time sensitive as delays carry economic and opportunity costs for universities and the health system alike [1]. Therefore, curricula should incorporate explicit strategies for the supervision and responsible use of these technologies, including guidelines for verification, documentation, and boundaries of use, a need that is underscored by evidence that the generational gap in AI tool mastery can strain teacher-student relationships and increase the risk of inappropriate use without adequate supervision frameworks [30,31]. Insufficient training could ultimately deepen disparities in care quality and limit Spain’s capacity to adopt clinical innovations at a pace comparable to other European countries [17].

This study has several limitations. First, reliance on publicly available information may not fully capture internal initiatives or unpublished plans. Second, the categorization of AI content depended on the terminology used in official documents, potentially resulting in inconsistent classifications. Third, because individual preconsensus classifications were not retained as separate records, a formal interrater reliability statistic (eg, Cohen kappa) could not be reported; however, the binary, mutually exclusive categories and the predefined >50% content threshold were designed to minimize subjectivity. Fourth, this cross-sectional design captured a single academic year (2025‐2026) and could not reflect the pace of curricular change. Finally, the authors’ use of AI-assisted tools, disclosed in the Acknowledgments section, could be perceived as reflecting a favorable stance toward AI adoption; however, data collection and classification were conducted independently of any AI tools, and all analytical decisions were made by the authors. Readers should be aware that the research topic itself may predispose toward a positive framing.

Future research should include direct surveys of academic coordinators at all 52 institutions to capture informal initiatives not visible in official documents. Longitudinal studies repeating this census at regular intervals would allow monitoring of curricular change over time. To enable systematic tracking, we propose the following four indicators: (1) proportion of universities offering at least one compulsory AI course, (2) mean ECTS specifically dedicated to AI per medical degree, (3) proportion of programs incorporating generative AI content, and (4) an interregional Gini coefficient of AI training distribution to quantify geographic equity. Implementation of such a framework would benefit from the involvement of the Spanish Ministry of Universities, National Agency for Quality Assessment and Accreditation (Agencia Nacional de Evaluación de la Calidad y Acreditación), medical school deans, and Spanish Society for Medical Education and Health Sciences (Sociedad Española de Educación Médica y de Ciencias de la Salud). International collaboration through organizations such as the International Association for Health Professions Education could further facilitate shared standards and translational research in digital health education.

### Research in Context

#### Evidence Before This Study

We searched PubMed, Scopus, and Web of Science databases up to July 2025 for studies on the inclusion of AI courses or formal AI training in undergraduate medical curricula. We found international initiatives describing the introduction of AI in medical education, mainly in North America and Asia, but these were often limited to individual universities or pilot projects. To our knowledge, no nationwide systematic evaluation of AI integration into undergraduate medical curricula in Spain had been reported.

#### Added Value of This Study

This study provides the first national overview of how AI is being integrated into undergraduate medical education in Spain. By analyzing the official curricula of all 52 universities offering medical degrees, we demonstrate that AI training is scarce, mostly elective, and carries minimal academic weight. We also identify striking regional disparities: while some regions such as Andalusia have implemented courses, others have none. This comprehensive analysis highlights the uneven and inconsistent adoption of AI in medical curricula.

#### Implications of All the Available Evidence

Our findings show that AI education in Spanish medical schools is at an early and unevenly distributed stage, with low ECTS credit weight and a predominance of elective courses. In light of international competency frameworks [13,14] and student-reported perceptions of unpreparedness [17], this limited provision may be insufficient to meet the evolving demands of AI-driven clinical practice. Considering international recommendations, the evidence supports the establishment of minimum standards, stronger national coordination, and systematic monitoring of AI training in medical curricula to ensure equitable and consistent preparation across regions.

### Conclusion

In conclusion, this study provides the first systematic assessment of artificial intelligenceAI integration in undergraduate medical education in Spain, revealing a landscape that warrants attention. AI training in medical curricula is still nascent and unevenly distributed, underscoring the need for updated educational policies to ensure that future health care professionals acquire competencies aligned with technological advances. Given the growing role of AI in clinical practice, this deficit in formal training may leave graduates less well equipped than peers in countries with more structured curricula. The main contribution is a comprehensive, census-based overview with an operationalized taxonomy (specific AI course and AI-similar course) that, unlike prior studies focused on isolated programs or non-standardized definitions, provides a consistent, reproducible framework reusable for longitudinal monitoring and international comparison, and a clear evidence base to inform curriculum reform, accreditation standards, and faculty development priorities.

## Supplementary material

10.2196/88511 Multimedia Appendix 1 Complete classification of Spanish universities offering a medical degree in the 2025-2026 academic year according to the presence of artificial intelligence–related training in its medical curriculum.

10.2196/88511 Checklist 1 STROBE checklist.

## Abbreviations

AI: artificial intelligence
ECTS: European Credit Transfer and Accumulation System
RUCT: Register of Universities, Centers and Degrees (Registro de Universidades, Centros y Títulos)
STROBE: Strengthening the Reporting of Observational Studies in Epidemiology
