# Supplementary material for: AI Integration in Spanish Undergraduate Medical Education: National Cross-Sectional Study
Source: JMIR Med Educ. 2026 Jun 8;12:e88511. doi: 10.2196/88511 (PMC13245843; doi:10.2196/88511)
Supplement: Multimedia Appendix 1 [file mededu-v12-e88511-s001.docx]

**Supplementary Table S1. Complete Classification of Spanish Universities Offering a Medicine Degree**

*Cross-sectional census of 52 Spanish universities offering an official Medicine degree, academic year 2025–2026, data collected July–September 2025. Universities are classified as offering a specific AI course, AI-similar content, or No AI-related training, according to the taxonomy described in the Methods section. Source URLs link to institutional websites where curricular information was publicly available at the time of data collection.*

| **University** | **Region** | **Type** | **Classification** | **Course Name** | **Source URL** |
| --- | --- | --- | --- | --- | --- |
| Autonomous University of Barcelona (UAB) | Catalonia | Public | Specific AI course | Artificial Intelligence and Health | https://www.uab.cat/web/estudiar/listado-de-grados/plan-de-estudios/plan-de-estudios-y-horarios/medicina-1345467893054.html?param1=1263281708763 |
| Camilo Jose Cela University (UCJC) | Madrid | Private | Specific AI course | Fundamentals of Artificial Intelligence in Healthcare | https://www.ucjc.edu/grados/medicina/ |
| Complutense University of Madrid (UCM) | Madrid | Public | Specific AI course | Practical Application of Generative Artificial Intelligence | https://medicina.ucm.es/asignaturas-optativas-081p https://medicina.ucm.es/file/806840-aplicacio%CC%81n-pra%CC%81ctica-de-la-inteligencia-artificial-generativa?ver |
| Fernando Pessoa University, Canary Islands (UFPC) | Canary Islands | Private | Specific AI course | Big Data and Artificial Intelligence in Medicine | https://ufpcanarias.es/titulacion/grado-en-medicina/ |
| Loyola Andalusia University (ULA) | Andalusia | Private | Specific AI course | Artificial Intelligence and Healthcare | https://www.uloyola.es/grados/grado-medicina#planestudios |
| University of Almeria (UAL) | Andalusia | Public | Specific AI course | Medical Informatics | https://www.ual.es/estudios/grados/presentacion/plandeestudios/3321 https://www.ual.es/guia_academica?idioma=CAS&anyaca=2025-26&asignatura=33214301 |
| University of Cordoba (UCO) | Andalusia | Public | Specific AI course | Documentation and Artificial Intelligence in Medicine. History of Medicine | https://www.uco.es/medicinayenfermeria/es/planificacion-ensenanza-med https://www.uco.es/eguiado/guias/2025-26/145004es_2025-26.pdf |
| University of Huelva (UHU) | Andalusia | Public | Specific AI course | Application of Big Data and Artificial Intelligence in Medicine | https://www.uhu.es/fenfe/sites/fenfe/files/2024-05/Planificacion-ense%C3%B1anzas-Grado-Medicina-UHU.pdf |
| University of Jaen (UJA) | Andalusia | Public | Specific AI course | New Clinical and Biomedical Information Technologies | https://uvirtual.ujaen.es/pub/es/informacionacademica/catalogoguiasdocentes/p/2024-25/7/106A |
| University of Lleida (UDL) | Catalonia | Public | Specific AI course | Artificial Intelligence in Medicine | https://graumedicina.udl.cat/export/sites/Medicina/ca/pla-formatiu/pla-estudis-guies-docents/PLA-destudis-per-cursos-i-assignatures-2025-26.pdf https://guiadocent.udl.cat/es/html/2025-26_100794 |
| Alfonso X El Sabio University (UAX) | Madrid | Private | AI-similar course | Digital Health | https://www.uax.com/titulaciones/grado-en-medicina |
| King Juan Carlos University (URJC) | Madrid | Public | AI-similar course | Introduction to Medicine: Medical Information and Documentation | https://servicios.urjc.es/listadoprofesorado/itinerario-formativo/grado-en-medicina/presencial/2000 |
| Rovira i Virgili University (URV) | Catalonia | Public | AI-similar course | New Technologies and Data Management | https://www.urv.cat/ca/estudis/graus/oferta/plans/ciencies-salut/medicina-grau/ https://www.urv.cat/ca/estudis/graus/oferta/plans/ciencies-salut/medicina-grau/#3 |
| University of Extremadura (UNEX) | Extremadura | Public | AI-similar course | Applied Medical Informatics | https://medicina.unex.es/titulaciones/0218/#tab-subjects |
| University of Oviedo (UNIOVI) | Asturias | Public | AI-similar course | .. | https://medicinaysalud.uniovi.es/documents/48520/4671527/GuiaDocenteGMEDIC013950073644869805761.pdf/594d4c47-dddf-f67c-c918-584e3d2ab54d?t=1750244266819 |
| University of Vic - Central University of Catalonia (UVic UCC) | Catalonia | Private | AI-similar course | Information and Communication Technologies and Health | https://www.uvic.cat/es/grado/medicina |
| Antonio de Nebrija University | Madrid | Private | No AI-related training | .. | https://www.nebrija.com/carreras-universitarias/grado-medicina/#planEstudios |
| Autonomous University of Madrid (UAM) | Madrid | Public | No AI-related training | .. | https://secretaria-virtual.uam.es/doa/consultaPublica/look[conpub]BuscarPubGuiaDocAs?entradaPublica=true&idiomaPais=es.ES&_centro=106&_planEstudio=652 |
| Catholic University of Murcia (UCAM) | Murcia | Private | No AI-related training | .. | https://www.ucam.edu/sites/default/files/public/grado-medicina/estructura-del-titulo-por-modulos.pdf |
| Catholic University of Valencia | Valencian Community | Private | No AI-related training | .. | https://www.ucv.es/oferta-academica/grados/grado-en-medicina/seccion/plan-de-estudios |
| CEU Cardenal Herrera University (UCHCEU) | Valencian Community | Private | No AI-related training | .. | https://www.uchceu.es/docs/estudios/plan-estudios/grado/medicina.pdf |
| CEU San Pablo University | Madrid | Private | No AI-related training | .. | https://www.uspceu.com/Portals/0/docs/oferta-academica/grados/grado-en-medicina/plan-2010/plan-estudios-med-17-18.pdf?ver=2018-01-26-080712-797 |
| European University of Madrid | Madrid | Private | No AI-related training | .. | https://universidadeuropea.com/grado-medicina-madrid/ |
| Francisco de Vitoria University (UFV) | Madrid | Private | **No AI-related training** | .. | https://www.ufv.es/estudiar-grado-medicina-madrid/#plan-2025 |
| International University of Catalonia (UIC) | Catalonia | Private | No AI-related training | .. | https://www.uic.es/sites/default/files/folleto-medicina-esp.pdf |
| Jaume I University (UJI) | Valencian Community | Public | No AI-related training | .. | https://www.uji.es/estudis/base/2021/graus/medicina-p17/?urlRedirect=https://www.uji.es/estudis/base/2021/graus/medicina-p17/&url=/estudis/base/2021/graus/medicina-p17/&p=2021/page-producto-documento-completo |
| Miguel Hernandez University (UMH) | Valencian Community | Public | No AI-related training | .. | https://www.umh.es/contenido/Estudios/:tit_g_132_M1_CAnt/datos_es.html?frm=pintaPlanEstudios |
| Public University of Navarra (UPNA) | Navarra | Public | No AI-related training | .. | https://www.unavarra.es/sites/grados/salud/medicina/plan-de-estudios.html#cCentralUPNA |
| San Jorge University | Aragon | Private | No AI-related training | .. | https://www.usj.es/estudios/grados/medicina/plan-de-estudios |
| U. de Sevilla (US) | Andalusia | Public | No AI-related training | .. | https://alojawebapps.us.es/fichape/Doc/HIM/HIMat_172.pdf |
| U. de Valencia (UV) | Valencian Community | Public | No AI-related training | .. | https://www.uv.es/uvweb/universidad/es/estudios-grado/oferta-grados/oferta-grados/grado-medicina-1285846094474/Titulacio.html?id=1285847387054&p2=2 |
| U. Pompeu Fabra (UPF) | Catalonia | Public | No AI-related training | .. | https://gestioacademica.upf.edu/doa/consultaPublica/look%5Bconpub%5DBuscarPubGuiaDocAs?entradaPublica=true&idiomaPais=ca.ES&_centro=336&_estudio=3363&_anoAcademico=2023 |
| University of Alcala (UAH) | Madrid | Public | No AI-related training | .. | https://www.uah.es/export/shared/es/estudios/.galleries/Archivos-estudios/GR/Unico/AG215_2_6_1_E_G215.pdf |
| University of Alicante (UA) | Valencian Community | Public | No AI-related training | .. | https://web.ua.es/es/grados/grado-en-medicina/plan-de-estudios.html#plan |
| University of Barcelona (UB) | Catalonia | Public | No AI-related training | .. | https://web.ub.edu/es/web/estudis/w/grado-G1046?subjects |
| University of Cadiz (UCA) | Andalusia | Public | No AI-related training | .. | https://medicina.uca.es/docencia/grado-en-medicina/planes-de-estudios-itinerario-curricular/ |
| University of Cantabria (UNICAN) | Cantabria | Public | No AI-related training | .. | https://web.unican.es/centros/medicina/estudios/asignaturas?pi=66&c=2024&ga=N |
| University of Castile-La Mancha (UCLM) | Castile-La Mancha | Public | No AI-related training | .. | https://www.uclm.es/albacete/medicina/gradomedicina/planestudios |
| University of Deusto | Basque Country | Private | No AI-related training | .. | https://www.deusto.es/es/inicio/estudia/estudios/grado/medicina/plan-de-estudios |
| University of Girona (UDG) | Catalonia | Public | No AI-related training | .. | https://www.udg.edu/es/estudia/oferta-formativa/oferta-dassignatures?idpla=3109G0111&anyacad=2025 |
| University of Granada (UGR) | Andalusia | Public | No AI-related training | .. | https://grados.ugr.es/medicina/docencia/plan-estudios#contenido0 |
| University of La Laguna (ULL) | Canary Islands | Public | No AI-related training | .. | https://www.ull.es/grados/medicina/plan-de-estudios/estructura-del-plan-de-estudios/ |
| University of Las Palmas de Gran Canaria (ULPGC) | Canary Islands | Public | No AI-related training | .. | https://www2.ulpgc.es/plan-estudio/4029/40/estructuraporcursos |
| University of Malaga (UMA) | Andalusia | Public | No AI-related training | .. | https://www.uma.es/centers/subjects_center/facultad-de-medicina/5365/ |
| University of Murcia (UMU) | Murcia | Public | No AI-related training | .. | https://www.um.es/web/estudios/grados/medicina/plan-guias |
| University of Navarra (UNAV) | Navarra | Private | No AI-related training | .. | https://www.unav.edu/web/grado-en-medicina/plan-de-estudios |
| University of Salamanca (USAL) | Castile and Leon | Public | No AI-related training | .. | https://www.usal.es/files/grados/planes/planes_estudio2022y210_medicina_0.pdf |
| University of Santiago de Compostela (USC) | Galicia | Public | No AI-related training | .. | https://www.usc.gal/es/estudios/grados/ciencias-salud/grado-medicina-2a-edicion |
| University of the Balearic Islands (UIB) | Balearic Islands | Public | No AI-related training | .. | https://www.uib.eu/Learn/estudis-de-grau/grau/medicina/GMED-P/assignatures.html |
| University of the Basque Country (UPV/EHU) | Basque Country | Public | No AI-related training | .. | https://www.ehu.eus/es/web/graduak/grado-medicina/creditos-y-asignaturas |
| University of Valladolid (UVA) | Castile and Leon | Public | No AI-related training | .. | https://med.uva.es/grado-en-medicina/seguimiento-titulo/ |
| University of Zaragoza (UNIZAR) | Aragon | Public | No AI-related training | .. | https://indo.unizar.es/proyecto/5861/ficha |

*Note: ".." indicates information not applicable or not publicly available. Classification criteria: "Specific AI course" = AI as primary topic (>50% of syllabus); "AI-similar course" = digital health or biomedical informatics courses referencing AI as secondary content (<50%); "No AI-related training" = no evidence of AI-related content in the degree plan or available course guides.*
